# Loss of the ClpXP Protease Leads to Decreased Resistance to Cell-Envelope Targeting Antimicrobials in *Bacillus anthracis* Sterne

**DOI:** 10.3389/fmicb.2021.719548

**Published:** 2021-08-23

**Authors:** Lang Zou, Christopher R. Evans, Vuong D. Do, Quinn P. Losefsky, Diem Q. Ngo, Shauna M. McGillivray

**Affiliations:** Department of Biology, Texas Christian University, Fort Worth, TX, United States

**Keywords:** Clp protease, ClpXP protease, ClpX, *Bacillus anthracis*, antibiotic resistance, antimicrobial peptides, cell wall

## Abstract

The ClpX ATPase is critical for resistance to cell envelope targeting antibiotics in *Bacillus anthracis*, however, it is unclear whether this is due to its function as an independent chaperone or as part of the ClpXP protease. In this study, we demonstrate that antibiotic resistance is due to formation of the ClpXP protease through construction of a ClpX complementation plasmid that is unable to interact with ClpP. Additionally, we genetically disrupted both *clpP* genes, *clpP1* and *clpP2*, found in *B. anthracis* Sterne and find that the loss of either increases susceptibility to cell envelope targeting antimicrobials, although neither has as strong of a phenotype as loss of *clpX* and neither *clpP* gene is essential for virulence in a *G. mellonella* model of infection. Lastly, we looked at changes to cell envelope morphology that could contribute to increased antibiotic sensitivity. We find no difference in cell charge or cell lysis, although we do see increased hydrophobicity in the Δ*clpX* strain, decreased cellular density and slightly thinner cells walls. We also see significant cell division defects in Δ*clpX*, although only when cells are grown in the mammalian cell culture medium, RPMI. We conclude that the intrinsic resistance of *B. anthracis* to cell wall active antimicrobials is dependent on formation of the ClpXP protease and that this could be due, at least in part, to the role of ClpX in regulating cell envelope morphology.

## Introduction

*Bacillus anthracis*, the etiological agent of the deadly disease Anthrax, is a gram-positive, spore forming bacterium. Previous work in our lab identified the *clpX* gene as essential for virulence in *B. anthracis* as well as resistance to antimicrobials that target the cell-envelope (McGillivray et al., 2009, 2012; Claunch et al., 2018). ClpX is a regulatory ATPase that functions in one of two ways, either as an independent chaperone or together with the caseinolytic peptidase (ClpP) to form the ClpXP protease (Wawrzynow et al., 1995; Baker and Sauer, 2011). As part of the ClpXP protease, ClpX recognizes proteins, unfolds them using ATP hydrolysis, and then feeds them into the proteolytic core created by ClpP, which degrades them. Although ClpX can function independently of ClpP, ClpP is dependent on its partner ATPase, either ClpX, ClpC or ClpA, to degrade anything larger than a small peptide (Baker and Sauer, 2011). Proteomic analyses in *E. coli* and *S. aureus* show that the ClpXP protease regulates numerus intracellular proteins including metabolic enzymes, stress response proteins, regulatory proteins, virulence factors, and damaged or misfolded proteins (Flynn et al., 2003; Kirsch et al., 2021). Because ClpXP controls the degradation of multiple proteins in the cell, it functions as a global regulator and loss of either component can have pleiotropic effects on the cell. Homologs of *clpX* and *clpP* are found in many bacterial species with functions in the stress response, cellular differentiation, cell morphology, and virulence (Frees et al., 2007).

Our studies with a genetic knockout of the *clpX* gene, Δ*clpX*, demonstrate that ClpX is important for maintaining resistance to the antimicrobial peptides LL-37 and nisin and the antibiotics penicillin and daptomycin in *B. anthracis* Sterne (McGillivray et al., 2009, 2012; Claunch et al., 2018). All of these antimicrobials interact with or target the cell wall and/or cell membrane. The antimicrobial peptides LL-37 and nisin target the cell membrane, forming pores that contribute to membrane damage and/or depolarization (Peschel and Sahl, 2006; Jensen et al., 2020). Modification of the bacterial cell wall is a known resistance mechanism to LL-37 and there is some evidence LL-37 may interfere with cell wall synthesis in *E. coli* (Peschel and Sahl, 2006; Sochacki et al., 2011). Daptomycin is linked to membrane damage and inhibition of cell wall synthesis (Müller et al., 2016) and bacterial resistance mechanisms are similar to those of cationic antimicrobial peptides such as LL-37 (Mishra et al., 2011). Recently, daptomycin has been reported to form a complex with peptidoglycan and lipid intermediates that inhibited cell wall synthesis and led to massive membrane rearrangements (Grein et al., 2020). Penicillin inhibits cell wall synthesis through interference with transpeptidation (Waxman and Strominger, 1983). Furthermore, previous studies have implicated loss of Clp proteins in abnormal cell wall structure and/or division defects (Weart et al., 2005; Frees et al., 2007, 2014; Jensen et al., 2019a,b).

It is less clear if the resistance against these cell-envelope targeting antimicrobials is dependent on ClpXP formation, independent chaperone-activity, or a combination of the two. We previously used a pharmacological inhibitor of ClpXP to demonstrate that formation of this protease is important for intrinsic resistance to cell-envelope active antibiotics (McGillivray et al., 2012). However, there is always the possibility of off-target effects with any inhibitor and use of this inhibitor does not rule out the possibility of ClpX chaperone activity in antibiotic resistance. In order to gain a better understanding of the roles of both ClpX and ClpP in mediating antibiotic resistance, we constructed a *clpX* complementation plasmid with a mutation at the ClpP-ClpX interaction site. We also explored the roles of the *clpP* genes in antibiotic resistance in *B. anthracis* Sterne. *B. anthracis* has two ClpP subunits, *clpP1* and *clpP2*, initially characterized in the related species, *Bacillus thuringiensis* (Fedhila et al., 2002). Lastly, we looked more closely at possible changes to cell envelope morphology that could explain some of the phenotypes we see. Specifically, we examined the role of cell charge, cell lysis, hydrophobicity, and cell wall thickness, which have been linked to resistance to cell-envelope targeting antimicrobials (Davies et al., 1996; Clarke et al., 2007; Nannini et al., 2010; Lather et al., 2016; Moravej et al., 2018). We find that ClpX-mediated antibiotic resistance is dependent on the formation of the ClpXP protease, that both *clpP* genes contribute to its activity, and that changes in cell-envelope morphology found in the Δ*clpX* mutant could contribute to increased susceptibility to cell-envelope targeting antibiotics.

## Materials and Methods

### Bacterial Strains and Growth Conditions

*Bacillus anthracis* Sterne strain 34F2 (pX01^+^, pX02^–^), which lacks the pXO2 encoded capsule genes and can be safely used in a biosafety level-2 laboratory, was cultured in Brain-Heart Infusion (BHI) media (Hardy Diagnostics, Santa Maria, CA, United States) at 37°C under aerobic conditions unless noted otherwise. Antibiotic selection was used at the following concentrations: *E. coli*: erythromycin 500 μg/ml and ampicillin (100 μg/ml); *B. anthracis*: erythromycin 5 μg/ml (Erm5) and spectinomycin (100 μg/ml) (all Sigma, St. Louis, MO, United States). 0.1 mM isopropyl β-d-1-thiogalactopyranoside (IPTG; IBI Scientific, Peosta, IA, United States) was added when inducing the expression plasmid pUTE657. Construction of *Streptococcus pyogenes* Δ*dltA*, *B. anthracis* strain Δ*clpX* and wild-type *clpX* complementation plasmid (Δ*clpX* + p*clpX*) along with the control strains of wild-type and Δ*clpX* containing the empty inducible plasmid pUTE657 were described previously (Kristian et al., 2005; McGillivray et al., 2009; Claunch et al., 2018). All other strains were constructed in this study.

### Construction of *clpX^*I*264*E*^* Plasmid

A substitution mutation at position 264 from isoleucine (ATT) to glutamic acid (GAA) of the *B. anthracis clpX* gene was made in the previously constructed *clpX* expression plasmid (Claunch et al., 2018) to yield the *clpX^*I*264*E*^* plasmid. To do this, site-directed mutagenesis (NEB, Ipswich, MA, United States) was performed using the primers described in Table 1 following manufacturer’s instructions. The plasmid was then sequenced to confirm mutagenesis was successful.

**TABLE 1:** Primers used in the study.

Name	Description	Sequence
Ba_ClpX I264E_Fwd	Site-directed mutagenesis	5′-TGAAAGGTAGAGGGATTTGGTTCTGAGAAG-3′
Ba_ClpX 1264E Rev	Site-directed mutagenesis	5′-CCAAGACGGCGTTTAATAATTG-3′
dltD QPCR Fwd	QPCR primers	5′-AGATCAAACAGGTGCAGCAG-3′
dltD QPCR_Rev	QPCR primers	5′-CAGGAACAGAGATGAAGAGTGG-3′
mprf QPCR Fwd	QPCR primers	5′-TTCGTTCCTCGGTCTTATCG-3′
mprf QPCR Rev	QPCR primers	5′-CTTGTCCTTCCGCTTGCTTC-3′
fusA QPCR Fwd	QPCR primers	5′-AAGCTGGTGGTGCTGAAGCAC-3′
fusA QPCR Rev	QPCR primers	5′-CCATTTGAGCAGCACCAGTGA-3′
clpP1_IM Fwd-*Hin*dIII	Insertional mutagenesis	5′-ATGCAAGCTTAACGCGCTTACGATATTTACTCTC-3′
clpP1_IM Rev-*Bam*HI	Insertional mutagenesis	5′-GCATGGATCCGCTTCACTGTTTGGAAGTGC-3′
clpP2_IM Fwd-*Hin*dIII	Insertional mutagenesis	5′-ATGCAAGCTTAGACCGTATTGTTATTATCGGTTCAGA-3′
clpP2_IM Rev-*Bam*HI	Insertional mutagenesis	5′-GCATGGATCCCACTATTTGGGAGTGCAAATCGT-3′
clpP1_EV Fwd-*Sal*I	Complementation plasmid	5′-ATGCGTCGACGGTTTCGTTTGACCTTTATTGAC-3′
clpP1_EV Rev-*Sph*I	Complementation plasmid	5′-TGCAGCATGCGATTCTAAACGAGCCGCTTCCG-3′
clpP2_EV Fwd-*Sal*I	Complementation plasmid	5′-ATGCGTCGACTGCTTTGCGTTGCATAACAATTATTG -3′
clpP2_EV Rev-*Sph*I	Complementation plasmid	5′-TGCAGCATGCACTTTACTCTCTAGCCTGCTTTCTG-3′
pHY3065 Fwd	Plasmid-specific primers	5′-ACGACTCACTATAGGGCGAATTGG-3′
pHY3260 Rev	Plasmid-specific primers	5′-GCGGATAACAATTTCACACAGG-3′
pUTE657 Fwd	Plasmid-specific primers	5′-GAACGTTGCTCGAGGGTAAATG-3′
pUTE657 Rev	Plasmid-specific primers	5′-GGTACGTACGATCTTTCAGCC-3′

### RNA Extraction and QPCR

Overnight cultures were pelleted and resuspended in 200 μl of water. A 400 μl of RLT buffer from the RNeasy Mini Kit (Qiagen) was added and the solution was transferred to a 2 ml vial containing 0.1 mm disruptor beads (BioExpress). Cells were mechanically disrupted using the Fast Prep-24 (MP Biomedicals) with two pulses at 6 m/s for 45 s and a 2-min rest on ice between pulses. The supernatant was transferred to a new tube, 250 μl of ethanol was added, and the sample was applied to the RNeasy column and extraction proceeded per the manufacturer’s protocol, including the extra RNA elution step. The RNA was treated with two rounds of DNase treatment using TURBO DNA-free Kit (Thermo Fisher Scientific). cDNA was made from the extracted RNA using High Efficiency cDNA Synthesis Kit (Thermo Fisher Scientific) per manufacturer’s protocol. SYBR Green PCR Mix (Thermo Fisher Scientific) was used for QPCR (Applied Biosystems 7500 Real-Time PCR System). Cycling conditions were 1 cycle of 50°C for 2 min then 95°C for 10 min followed by 40 cycles of 95°C for 15 s and 60°C for 1 min. Amplification efficiency for all primers is near 100% (± 10%) under the conditions used. Relative expression of genes was calculated using the Livak method (Livak and Schmittgen, 2001) and normalized to the reference gene *fusA*. Experiments were performed with RNA from three independent preparations.

### Construction of *B. anthracis clpP1* and *clpP2* Insertional Mutants

To disrupt the *clpP1* and *clpP2* genes by insertional mutagenesis, an approximately 300 bp internal fragment of each was amplified via PCR using primers engineered with 5′ extensions containing restriction sites (described in Table 1). The resulting amplicon was subcloned into the temperature-sensitive plasmid pHY304 (all enzymes NEB, Ipswich, MA, United States) and transformed into electrocompetent MC1061F *Escherichia coli* (Lucigen, Middleton, WI, United States). Plasmids were extracted via miniprep (IBI Scientific, Peosta, IA, United States) and transformed into methylation deficient *E. coli* strain GM2163. GM2163 grown plasmids were extracted, transformed into electrocompetent *B. anthracis* Sterne as previously described (Koehler et al., 1994), and plated under Erm5 selection at 30°C. *B. anthracis* Sterne containing the insertional mutant plasmids were passed two times at 37°C in BHI-Erm5 to force plasmid integration. Integration into the bacterial chromosome was confirmed by PCR using the pHY304-Fwd primer and either clpP1_EV Rev-*Sph*I or clpP2_EV Rev-*Sph*I (Table 1).

### Construction of *clpP1* and *clpP2* Complementation Plasmids

Complementation plasmids were constructed by amplifying the complete *clpP1* or *clpP2* sequences using primers found in Table 1. The amplicons were then subcloned into pUTE657 (Pflughoeft et al., 2011) using *Sal*I and *Sph*I restriction endonucleases and transformed into MC1061F electrocompetent *E. coli* (Lucigen, Middleton, WI, United States). Successful transformation was confirmed using the pUTE657 plasmid specific primers (Table 1) and the correct full length *clpP1* and *clpP2* sequences were confirmed by sequencing. Plasmids were passaged through GM2163, extracted, transformed into Δ*clpP1* or Δ*clpP2 B. anthracis* Sterne, and grown at 37°C under spectinomycin selection. As controls, the empty inducible pUTE657 plasmid was also transformed into *B. anthracis* Δ*clpP1* or Δ*clpP2*.

### Growth Curves

Overnight cultures of wild-type *B. anthracis* Sterne, Δ*clpX*,Δ*clpP1* insertional mutant and Δ*clpP2* insertional mutant were diluted 1:20 and grown to early log phase at an optical density (OD) of 0.4 at 600 nm wavelength. Log phase cultures were then diluted 1:100 in fresh media, either BHI, Roswell Park Memorial Institute 1640 (RPMI) medium (Corning, Corning NY, United States) supplemented with 5% Luria–Bertani (LB) medium (Hardy Diagnostics, Santa Maria, CA, United States, hereafter known as RPMI-5% LB, or Mueller–Hinton Broth II (BD, Franklin Lakes, NJ, United States) supplemented with 50 μg/ml calcium hereafter known as CA-MHB. Growth was monitored over an 8-h period by measuring the OD of cultures in 1-h increments.

### Antimicrobial Susceptibility Assays

Overnight cultures were diluted and grown to early log phase at an OD of 0.4 at 600 nm wavelength in BHI. Log phase cultures were diluted 1:20 (∼6 × 10^5^ cfu/ml) in a final volume of 200 μl with antibiotics at concentrations indicated. Assays using nisin, vancomycin, and penicillin (all Sigma) were performed in BHI, assays using LL-37 (Anaspec, Fremont, CA, United States) were performed in RPMI-5% LB, and assays using daptomycin (Cubist pharmaceuticals, Lexington, MA, United States) were performed in CA-MHB at indicated concentrations. For testing LL-37 and daptomycin, cultures were pelleted, washed and re-suspended in an equivalent volume of RPMI-5% LB or CA-MHB. Assays were incubated overnight in flat bottom 96-well plates for 16–20 h at 37°C under static conditions except for the daptomycin assays, which were incubated in round bottom 96-well plates under shaking conditions, and the LL-37 assays, which were incubated between 24–36 h because of slow growth in RPMI. Following incubation, the OD of each well was measured at 600 nm wavelength and the minimum inhibitory concentration (MIC) was noted. 0.1 mM IPTG was included in the overnight culture, log phase culture and assay media for assays testing strains with the complementation plasmid in order to induce expression in pUTE657.

### *Galleria mellonella* Assay

*G. mellonella* were obtained from Rainbow Mealworms^1^, stored at 4°C to induce torpidity, and used within 5 days of receipt. Larvae weighing 190–200 mg were injected through the posterior cuticle using an automated pump (New Era Pump Systems NE-500, Farmingdale, NY, United States) and a 27-gauge needle. Bacterial cultures were grown overnight, diluted 1:20, and grown to early log phase (OD 0.4) in BHI. Once OD 0.4 was reached, bacteria were washed and suspended in PBS at a 1:2 dilution. Larvae were injected with 10 μl of the 1:2 dilution (approximately 5 × 10^4^ cfu/larvae) and starting amounts were confirmed through colony counts. After injection, larvae were observed at room temperature to ensure they recovered from injection and were transferred to an incubator at 37°C. Surviving larvae were counted at 24, 48, and 72-h post injection.

### Cell Charge

Bacterial cultures (*S. pyogenes*, stationary phase; *B. anthracis* log phase) were washed in 20 mM HEPES buffer (pH 7.5), then centrifuged and resuspended to an optical density of 0.1 at 600 nm. They were then incubated with 10 μg/mL poly-L-lysine for 30 min at room temperature in a total volume of 0.5 mL. After incubation, the samples were centrifuged at 16,000 rpm for 3 min. The fluorescent excitation of the supernatant was measured at 485 nm.

### Hydrophobicity Assay

Bacteria were grown overnight at 37°C in BHI, pelleted and washed twice with PBS and resuspended in 200 μl PBS. The concentrated bacteria were then added to 3 ml PBS to reach an OD of 0.5 at 600 nm wavelength (A0). 1 ml of n-hexadecane (Sigma-Aldrich, St. Louis, MO, United States) was then added to each tube. After vigorous vortexing, phases were allowed to separate for 30 min at room temperature and the OD600 nm of the lower aqueous phase was measured (A1). The percentage of hydrophobicity was calculated as follows: hydrophobicity (%) = [1-(A1/A0)] × 100. This was normalized to wild-type *B. anthracis* Sterne by dividing the percentage hydrophobicity for each strain by the hydrophobicity percentage of the wild type.

### Buoyancy Assay

A concentration gradient was created by layering 1 ml each of 70, 60, and 50% solutions of percoll (Sigma-Aldrich, St. Louis, MO, United States), with 50% as the top layer in glass test tubes. Bacteria were grown overnight at 37°C in BHI supplemented with 0.1 mM IPTG and 1 ml of each overnight culture was added to the top of the Percoll gradient. Tubes were centrifuged at 620 rpm at 4°C for 1 h using the Allegra^TM^ 6R centrifuge (Beckman Coulter, Pasadena, CA, United States) and the distance the bacteria traveled from the top of the gradient was measured.

### Transmission Electron Microscopy (TEM)

Unless otherwise noted, chemicals were purchased from Electron Microscopy Sciences (Hatfield, PA, United States). Bacterial cultures were grown overnight in BHI or RPMI-5% LB and washed in PBS. Samples were resuspended and fixed in 2.5% glutaraldehyde (Sigma-Aldrich, St. Louis, MO, United States) for 1 h at room temperature. Bacterial pellets were then washed in PBS three times. The samples were incubated in 1% osmium tetroxide for 1.5 h on ice. Osmium tetroxide was removed and samples were dehydrated with a series of 10-min ethanol washes at the following concentrations (in order): 30, 50, 70, 85, 95, and 100%, repeating the wash in 100% ethanol three times. Samples were then incubated in 100% propylene oxide for 15 min. Next, the samples were incubated 24 h at room temperature in 50% propylene oxide/50% Embed plastic (10 g Embed 812, 8 g DDSA, 4 g NMA, and 0.6 g DMP 30). After the 24-h incubation, the samples were embedded in 100% plastic and kept at 60° C for 72 h. 90 nm sections were made and placed on copper grids. Sections were stained in uranyl acetate stain (2 g of uranyl acetate in 100 mL of 50% acetone) for 4 min. Sections were washed in 50% acetone, then stained in lead citrate for 4 min. Lead citrate stain was made by dissolving 0.08 g of lead citrate (Ted Pella, Redding, CA, United States) in 18 mL of previously boiled water and 2 mL of 1.0 M NaOH. Sections were washed in 0.05 M NaOH, followed by a wash in previously boiled water and dried on filter paper. Images were taken at 200 kV using a JEOL 2100 Transmission Electron Microscope. To quantitate cell wall thickness, images of bacterial cross-sections were taken at 6000× magnification. Cell wall thickness measurements were collected by taking a linear profile across the cell membrane and cell wall, resulting in a histogram of electron densities. The distance from the low electron beam counts of the cell membrane to the high electron beam counts found in the cell wall was then measured.

### Statistics

GraphPad Prism (San Diego, CA, United States) was used for all statistical analysis. To correct for error caused by multiple comparisons in the survival curves, the Bonferroni corrected threshold was computed (level of statistical significance was set as 0.05 and divided by the number of comparisons being made).

## Results

### Formation of the ClpXP Protease Is Essential for Maintaining Antibiotic Resistance

Interaction between ClpX and ClpP is dependent on the presence of a 3 amino acid sequence, the IGF tripeptide. Mutations in this sequence eliminate the ability of ClpX to bind to ClpP without affecting its other functions (Baker and Sauer, 2011). In *S. aureus* a substitution mutation was made in ClpX at position 265 to change isoleucine to glutamic acid, which prevented formation of the ClpXP protease (Stahlhut et al., 2017). We constructed an analogous mutation in *B. anthracis* ClpX at position 264 from isoleucine (ATT) to glutamic acid (GAA) in our previously constructed *clpX* expression plasmid (Claunch et al., 2018) to create the plasmid p*clpX^*I*264*E*^*. After confirming the mutagenesis by sequencing, we transformed Δ*clpX B. anthracis* with p*clpX^*I*264*E*^* to generate Δ*clpX* + p*clpX^*I*264*E*^*.

**FIGURE 1:**
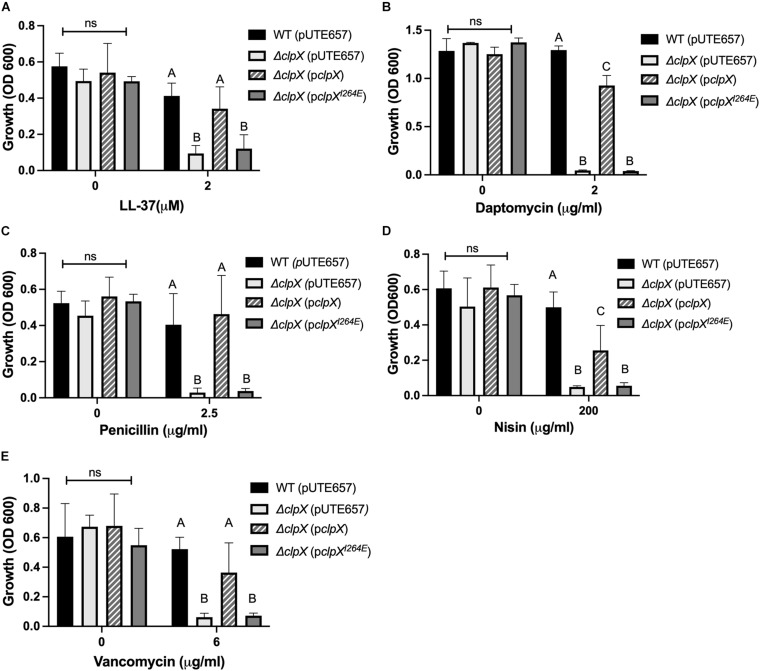
Formation of the ClpXP protease is necessary to maintain antimicrobial resistance in *B. anthracis* Sterne. Overnight growth of wild-type *B. anthracis* Sterne (WT), Δ*clpX* mutant containing the empty plasmid (pUTE657), and Δ*clpX* complemented with either the wild-type *clpX* gene (p*clpX*) or the *clpX* gene containing the I264E point mutation (p*clpX^*I*264*E*^*) in media containing either **(A)** LL-37, **(B)** daptomycin, **(C)** penicillin, **(D)** nisin, or **(E)** vancomycin. Data are presented as mean ± SD and assays were repeated at least three independent times. Different numbers represent statistically significant differences (*p* < 0.05) and ns indicates no statistical difference within each treatment group as determined by one-way ANOVA followed by Tukey–Kramer *post hoc* analysis.

To determine the effects of losing the ClpXP protease on antimicrobial susceptibility, we performed antimicrobial susceptibility assays with antimicrobials that target the cell envelope including two antimicrobial peptides, LL-37 and nisin, and three antibiotics, daptomycin, penicillin and vancomycin. We have previously shown that Δ*clpX* is more susceptible to LL-37, nisin, daptomycin and penicillin (Claunch et al., 2018) and, as expected, we find a significant difference in growth between the wild-type *B. anthracis* and Δ*clpX* strain with these 4 antimicrobials as well as with vancomycin (Figures 1A–E). This growth defect is rescued in the strain complemented with wild-type *clpX* (p*clpX*), however, when we complemented with p*clpX^*I*264*E*^*, the levels of growth were the same as Δ*clpX* in all antimicrobials tested. We conclude that ClpX-mediated antibiotic resistance to cell envelope targeting antimicrobials is dependent on formation of the ClpXP protease rather than independently through ClpX chaperone activity.

### Both *clpP1* and *clpP2* Contribute to Antibiotic Resistance

**FIGURE 2:**
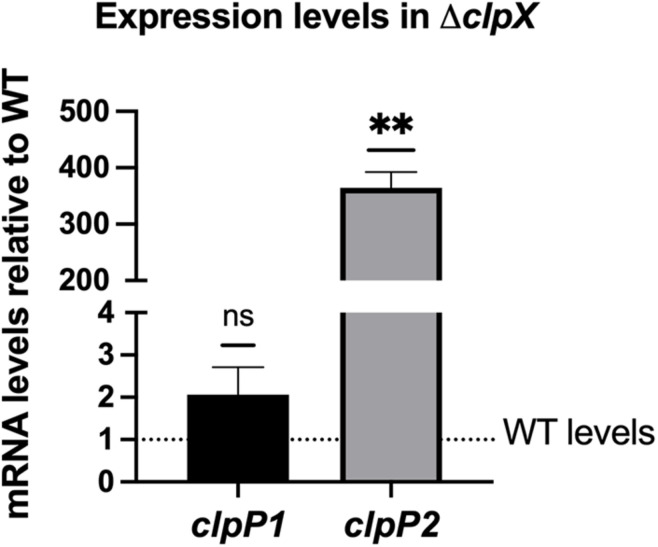
*B. anthracis clpP2* expression is regulated by ClpX. Expression levels of *clpP1* and *clpP2* in Δ*clpX B. anthracis* Sterne are shown relative to expression in the wild-type (WT) strain (dotted line). Data are presented as mean ± SD from three independent RNA preparations. Statistical significance determined by a one-sample *t*-test; **p < 0.01 or non-significant (ns).

**FIGURE 3:**
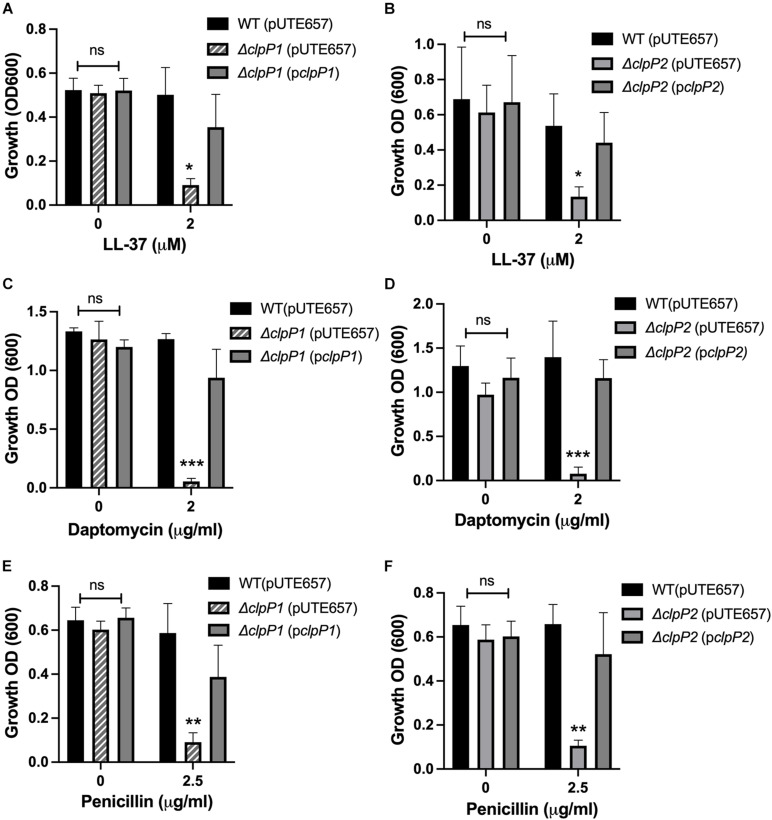
Both *clpP1* and *clpP2* genes contribute to antimicrobial resistance. Overnight growth of wild-type *B. anthracis* Sterne (WT), Δ*clpP1* or Δ*clpP2* containing the empty inducible plasmid (pUTE657), and Δ*clpP1* or Δ*clpP2* complemented with either the wild-type *clpP1* (p*clpP1*) or *clpP2* (p*clpP2*) genes in media containing **(A,B)** LL-37, **(C,D)** daptomycin or **(E,F)** penicillin. Data are presented as mean ± SD and assays were repeated at least 3 independent times. Statistical significance determined by a one-way ANOVA followed by Tukey-Kramer *post hoc* test; *p < 0.05; **p < 0.01; ***p < 0.001 or non-significant (ns) from WT levels within each treatment group.

Unlike most gram-positive bacteria, *B. anthracis* has two highly similar ClpP subunits, ClpP1 and ClpP2, with 66.32% of the amino acid sequences identical and 88.08% of the amino acid sequence identical or highly similar by ClustalW alignment (McGillivray et al., 2012). As is the case in *B. thuringiensis* (Fedhila et al., 2002), *clpP1* is organized as a monocistronic unit whereas *clpP2* is likely part of a two-gene operon with the first gene encoding an RNA-polymerase Sigma-70 factor. Regulation of the two *clpP* genes is strikingly different. Expression of *clpP2* but not *clpP1* is controlled by *clpX*, with *clpP2* levels increasing over 300-fold in the Δ*clpX* mutant relative to wild-type as seen by QPCR in stationary phase cultures (Figure 2). Similar results were seen in a previous microarray analysis using exponential phase cultures where both *clpP2* and sigma-70 were upregulated nearly 200-fold in the Δ*clpX* mutant (Claunch et al., 2018). This indicates that loss of *clpX* results in increased *clpP2* expression in either growth phase and further supports the regulation of *clpP2* and sigma-70 as a polycistronic operon. In *B. thuringiensis*, the two ClpP subunits control different regulatory pathways (Fedhila et al., 2002); therefore, we determined the role of each subunit in antibiotic resistance in *B. anthracis*. To do this, we individually disrupted each of the *clpP* genes in *B. anthracis* Sterne through insertional mutagenesis and created complemented strains bearing the respective wild-type genes on the inducible expression plasmid, pUTE657. As a control, we also transformed the empty pUTE657 plasmid into the Δ*clpP1* and Δ*clpP2* mutants to create Δ*clpP1* + pUTE657, Δ*clpP1* + p*clpP1*, Δ*clpP2* + pUTE657, and Δ*clpP2* + p*clpP2.* Both *clpP* mutants are more susceptible to LL-37, daptomycin and penicillin than the wild-type strain and the complemented strains reversed this phenotype (Figure 3). We next tried swapping complementation plasmids by pairing Δ*clpP1* with p*clpP2* and Δ*clpP2* with p*clpP1* to test whether loss of resistance is due to a reduction in total ClpP levels rather than loss of the specific ClpP1 or ClpP2 subunits. We see no complementation in penicillin with the swapped plasmids (Supplementary Figure 1) indicating that both *clpPs* contribute to resistance.

**FIGURE 4:**
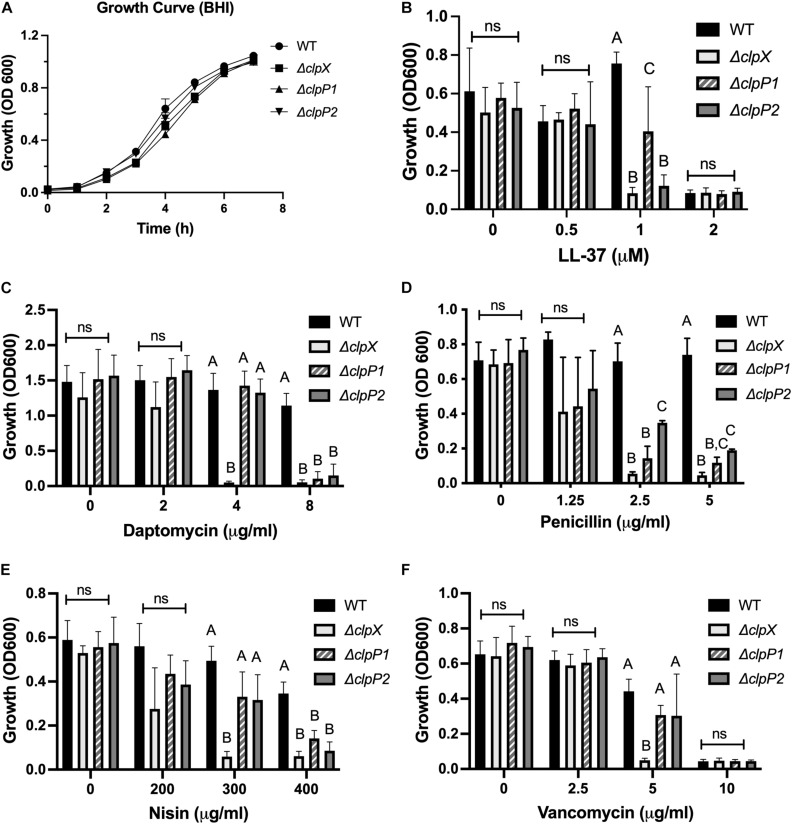
ClpX, ClpP1, and ClpP2 contribute to antimicrobial resistance to varying degrees. Growth of wild-type *B. anthracis* Sterne (WT), Δ*clpX*,Δ*clpP1* or Δ*clpP2* in **(A)** BHI alone or growth overnight in media containing either **(B)** LL-37, **(C)** daptomycin, **(D)** penicillin, **(E)** nisin, or **(F)** vancomycin. Statistical significance determined by one-way ANOVA followed by Tukey–Kramer *post hoc* analysis. Different numbers represent statistically significant differences (*p* < 0.05) and ns means no statistical difference within each treatment group. Data are presented as mean ± SD. The growth curve was repeated twice; all other assays were repeated at least three independent times.

We next compared the phenotypes of all three of our mutants, Δ*clpX*,Δ*clpP1*, and Δ*clpP2* in response to cell envelope targeting antimicrobials to assess relative contributions. We first performed growth curves over 8 hrs to determine whether the mutants grew at significantly different rates in any of the media used in our assays. Minimal differences in growth rates are seen at 37°C in BHI (Figure 4A) with similar results in RPMI-5% LB and CA-MHB (Supplementary Figures 2A,B). We next compared their resistance to the same cell-envelope targeting antimicrobials we tested in Figure 1. The MICs can be seen in Table 2 and growth at sub-MIC levels are shown in Figure 4. The LL-37 MIC is 2 μM for both wild-type and Δ*clpP1*, however the growth of Δ*clpP*1 is significantly impaired at 1 μM relative to wild-type (Figure 4B). In contrast, no growth is seen with Δ*clpX* or Δ*clpP2* at 1 μM. A similar trend is seen with nisin (Figure 4E). The MICs for penicillin and daptomycin are lower for all three mutants with Δ*clpX* showing increased susceptibility relative to both Δ*clpP1* and Δ*clpP2* (Figures 4C,D). Small differences between the *clpP* mutants can be seen in LL-37 and penicillin. While both have increased susceptibility relative to wild-type, a slightly weaker phenotype is seen with Δ*clpP1* in LL-37 and Δ*clpP2* with penicillin. Only Δ*clpX* showed increased susceptibility to vancomycin (Figure 4F). We conclude that loss of *clpX* has the most profound effect on antibiotic resistance, although both *clpP1* and *clpP2* are also necessary for resistance against the antimicrobials tested except vancomycin. We also find that although *clpP1* and *clpP2* are both necessary for full resistance, there may be slight differences in relative contribution.

**TABLE 2:** MICs of wild-type *B. anthracis* Sterne and the Δ*clpX*, Δ*clpP1*, and Δ*clpP2* mutants.

Strain	LL-37	Penicillin	Daptomycin	Nisin	Vancomycin
WT	2 μM	40 μg/ml	16 μg/ml	800 μg/ml	10 μg/ml
Δ*clpX*	1 μM	2.5 μg/ml	4 μg/ml	300 μg/ml	5 μg/ml
Δ*clpP1*	2 μM	10 μg/ml	8 μg/ml	800 μg/ml	10 μg/ml
Δ*clpP2*	1 μM	10 μg/ml	8 μg/ml	400 μg/ml	10 μg/ml

### ClpP1 and ClpP2 Are Not Essential for Virulence in *G. mellonella*

**FIGURE 5:**
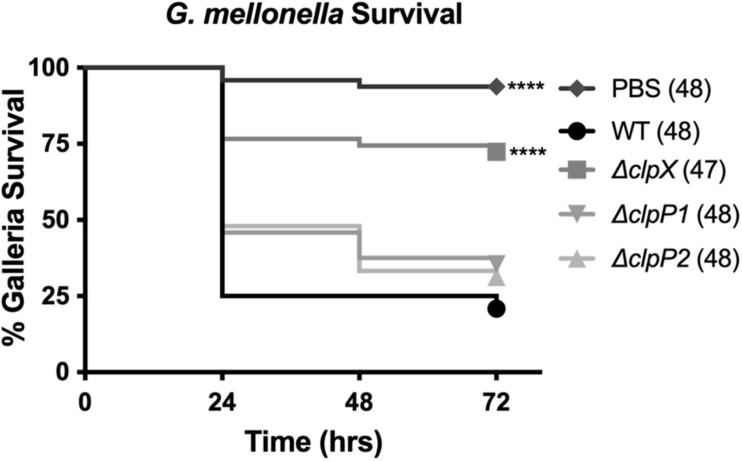
Loss of *clpP1* or *clpP2* does not attenuate virulence in *G. mellonella*. Survival of larvae injected with PBS or wild-type *B. anthracis* Sterne (WT), Δ*clpX*,Δ*clpP1* or Δ*clpP2.* Each infection was repeated four independent times and total number of larvae for each condition are indicated in parentheses. Significantly different survival from larvae injected with WT represented by ****p < 0.0001 by the Mantel-Cox log rank test.

ClpX is essential for virulence in *B. anthracis*, even in the fully virulent, encapsulated strain (McGillivray et al., 2009). To determine the role of the ClpP subunits in virulence, we used the caterpillar larvae of *G. mellonella*, an invertebrate infection model that shares many similarities to the mammalian innate immune system and that has been previously shown to be a reliable animal model for *B. anthracis* Sterne (Malmquist et al., 2019). We compared *G. mellonella* survival after injection with either PBS or the *B. anthracis* Sterne strains (Figure 5). Nearly all larvae (> 90%), survived injection with PBS alone while only 21% survived injection with wild-type *B. anthracis* Sterne. In contrast, 72% of larvae survived injection with Δ*clpX*, a highly significant difference from the wild type. The *G. mellonella* survival rates after injection with Δ*clpP1* and Δ*clpP2* were nearly identical (35 and 31% respectively) but this was not significantly different than the wild-type curve. Therefore, we conclude neither Δ*clpP1* nor Δ*clpP2* is as attenuated as Δ*clpX* in an animal model of infection and that loss of neither individual *clpP* subunit by itself is enough to affect virulence.

### Δ*clpX* Exhibits Differences in Cell Envelope Composition

**FIGURE 6:**
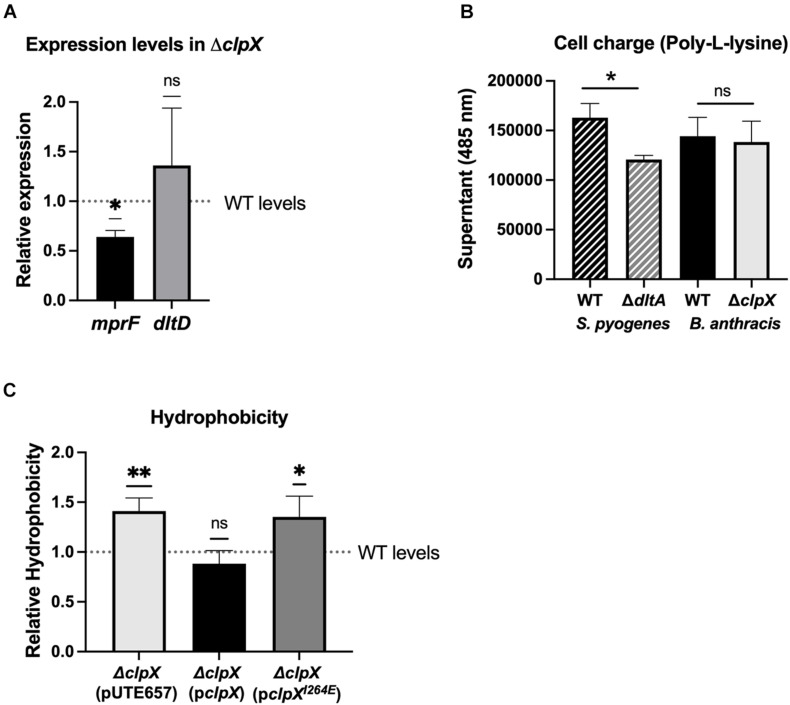
Δ*clpX* has increased hydrophobicity but no changes in cell charge. **(A)** Expression levels of the *mprf* and *dltD* genes in Δ*clpX* relative to wild-type (WT) levels (dotted line). *p < 0.05 as determined by a one-sample *t*-test. **(B)** Amount of cationic poly-L-lysine that sequesters in the supernatant after incubation with WT *S. pyogenes*, WT *B. anthracis* Sterne or their respective mutants Δ*dltA* and Δ*clpX*. *p < 0.05 as determined by unpaired *t*-test between WT and mutant of each species. **(C)** Amount of the Δ*clpX* mutant containing the empty plasmid (pUTE657), and Δ*clpX* complemented with either the wild-type *clpX* gene (p*clpX*) or the *clpX* gene containing the I264E point mutation (p*clpX^*I*264*E*^*) that sequesters in n-hexadecane relative to the WT strain (dotted line). *p < 0.05, **p < 0.01 as determined by a one-sample *t*-test. Data presented as mean ± SD from at least three independent experiments.

Given that Δ*clpX* is consistently attenuated in its response to cell envelope targeting antimicrobials and that loss of Δ*clpX* has been linked to disruptions in cell wall morphology in other bacterial species (Frees et al., 2007), we examined whether changes in the cell envelope in *B. anthracis* Sterne occurred upon loss of *clpX*. We first looked at the relative surface charge of the cell since increasing the positive charge of the cell wall is a known resistance mechanism for cationic molecules. The dlt*ABCD* operon increases the positive charge by modifying D-alanine in teichoic acids and *mprF* incorporates l-lysine into phosphatidylglycerol in the cell envelope and loss of either increases susceptibility to LL-37 and daptomycin (Fisher et al., 2006; Peschel and Sahl, 2006; Samant et al., 2009; Bayer et al., 2013). We measured *mprF* and *dltD* gene expression in the Δ*clpX* mutant and found that there was a slight but statistically significant decrease in *mprF* expression although no significant change in *dltD* (Figure 6A). We next measured cell charge by assessing how well fluorescently labeled, positively charged poly-L-lysine binds to the bacterial cell. The more positively charged the cell wall, the more the poly-L-lysine will sequester in the supernatant rather than bind to the bacterial pellet. As a control, we included the *S. pyogenes* Δ*dltA* mutant, which is more negatively charged (Kristian et al., 2005). As expected, Δ*dltA S. pyogenes* had less poly-L-lysine found in the supernatant relative to the wild-type strain, but no difference is seen between wild-type and Δ*clpX B. anthracis* Sterne (Figure 6B). To confirm this result, we also performed this same assay with cationic cytochrome c but again see no difference between WT and Δ*clpX* (Supplementary Figure 3). Therefore we conclude that despite the slightly decreased *mprf* expression in the Δ*clpX* mutant, there are no differences in cell charge between wild-type and Δ*clpX B. anthracis* Sterne in the assay conditions tested.

We next assessed whether hypoosmotic shock would reveal changes in cell wall integrity. We suspended wild-type and Δ*clpX* in pure water to assess osmotic lysis, but we see no difference in the rate of cell lysis (Supplementary Figure 4A). In a previous study, we found no change in autolytic activity between wild-type and Δ*clpX* (Claunch et al., 2018). We repeated those assays, but with an additional hypoosmotic shock (incubation for 10 min in ice cold water) that preceded induction of autolytic activity in 0.05% Triton X. Once again, no difference is seen in the rate of cell lysis between wild-type and Δ*clpX* (Supplementary Figure 3B). Therefore, if there is any difference in the structural integrity of the cell wall, it is not discernible in our assays.

We next looked at overall hydrophobicity of the cell envelope as cell wall components such as lipoteichoic acid are known to contribute to hydrophobicity (Courtney et al., 2009) and hydrophobicity has been linked to sensitivity to cell-envelope targeting antimicrobials (Davies et al., 1996; Clarke et al., 2007; Lather et al., 2016). The relative hydrophobicity of each strain was determined by measuring how readily the strains adhered to n-hexadecane, a highly hydrophobic compound. We find that the Δ*clpX* + pUTE657 and the Δ*clpX* + *pclpX^*I*264*E*^* strains showed significantly greater adhesion to n-hexadecane compared to the wild-type or the Δ*clpX* + p*clpX* complemented strain (Figure 6C), suggesting that loss of *clpX* increases cell envelope hydrophobicity. We also tested the Δ*clpP1* and Δ*clpP2* mutants but found no difference relative to wild-type (Supplementary Figure 5). This likely reflects the fact that neither individual *clpP* mutant has a phenotype as strong as Δ*clpX*.

**FIGURE 7:**
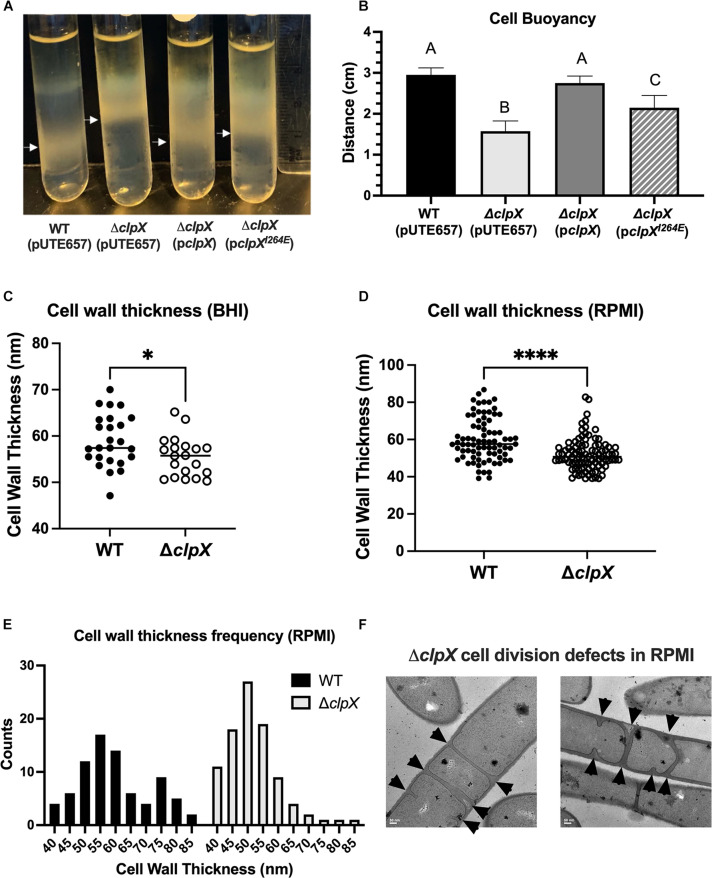
Loss of *clpX* results in changes to cell density and cell wall morphology. **(A,B)** Travel of wild-type *B. anthracis* Sterne (WT), Δ*clpX* mutant containing the empty inducible plasmid (pUTE657), and Δ*clpX* complemented with either the wild-type *clpX* gene (p*clpX*) or the *clpX* gene containing the I264E point mutation (p*clpX^*I*264*E*^*) through a percoll density gradient **(A)** representative image with white arrows marking final position **(B)** quantification of the distance traveled through the density gradient. Data represents mean ± SD of four independent experiments. Different numbers represent statistically significant differences (*p* < 0.05) by one-way ANOVA followed by Tukey–Kramer *post hoc* analysis. **(C,D)** Cell wall thickness measurements taken from TEM images of WT and Δ*clpX* transverse cross-sections after bacteria were grown in **(C)** BHI or **(D)** RPMI + 5% LB with *p < 0.05; ****p < 0.0001 by unpaired t-test. Each dot represents the measurement of the cell wall from a different cell. **(E)** Histogram of cell wall thickness measurements. **(F)** TEM images of Δ*clpX* grown in RPMI + 5% LB. Arrowheads indicate septum formation. Scale bar at 50 nm.

Lastly, we investigated whether there were changes in cell wall thickness that could account for differences in antibiotic susceptibility as this has been linked to antibiotic resistance in vancomycin and daptomycin (Nannini et al., 2010). Cell wall thickness is one of several factors that contribute to overall cell density, so we initially performed a buoyancy assay, which measures the ability of bacteria to penetrate a density gradient (Makinoshima et al., 2002). We found that Δ*clpX* is significantly less dense than the wild-type strain and that this phenotype is reversed by the strain complemented with wild-type p*clpX* but only partially with the mutant p*clpX^*I*264*E*^* (Figures 7A,B). In order to examine the actual cell wall, we used TEM to compare the thickness of the cell wall in transverse cross sections of the wild type and Δ*clpX*. We find a small but statistically significant decrease in the thickness of the Δ*clpX* cell wall when the cells are grown in BHI, the most common medium we use. However, we also frequently use the mammalian cell culture medium RPMI in our assays with LL-37, primarily because the antimicrobial peptide works more efficiently in that medium than BHI. Media components such as high salt interfere with LL-37 activity directly, but media composition also influences bacterial cell morphology and that can alter bacterial resistance to AMPs (Dorschner et al., 2006). Since RPMI could influence cell morphology and it is more likely to be analogous to host conditions than BHI, we grew the bacteria in RPMI-5% LB and examined cell wall structure. We find that growth in RPMI-5% LB results in even thinner cell walls in Δ*clpX* (Figures 7C,D). Whereas the cell wall thickness of wild-type *B. anthracis* Sterne grown in BHI or RPMI was nearly identical with an average thickness of 59 ± 5.6 nm or 59.9 ± 11.4 nm respectively, Δ*clpX* grown in BHI has an average thickness of 55.5 ± 4.3 nm while in RMPI it is 52 ± 8.7 nm. Besides the difference in average cell wall thickness, the range and variation in cell wall thickness was different between wild-type and Δ*clpX* grown in RPMI-5% LB. When plotted in a histogram, cell wall thickness measurements from wild-type bacteria grown in RPMI-5% LB show a bimodal curve with a peak at 55 nm and a peak at 75 nm whereas cell wall thickness measurements of Δ*clpX* bacteria show a single peak at 50 nm (Figure 7E). This indicates that not only are the cell walls thicker in wild-type *B. anthracis* Sterne than Δ*clpX*, they also exhibit more variation when grown in RPMI-5% LB.

We also observed abnormal cell division defects in Δ*clpX* with multiple septa forming at the division sites (arrowheads, Figure 7F). Interestingly, this is only observed when Δ*clpX* is grown in RPMI-5% LB but not when wild-type *B. anthracis* Sterne is grown in RPMI-5% LB nor when Δ*clpX* is grown in BHI (data not shown). After observing these differences in cell wall morphology after growth in RPMI, we repeated our cell charge and autolysis experiments with WT and Δ*clpX* grown in RPMI-5% LB, but we see no changes in activity from cells grown in BHI (data not shown). Cell division is mediated by FtsZ, a homolog of tubulin, through formation of the Z-ring at division sites. In *Bacillus subtilis*, ClpX prevents FtsZ polymerization and Z-ring formation and appears to be important for maintaining levels of unassembled FtsZ (Weart et al., 2005). We tried overexpressing FtsZ in wild-type *B. anthracis* Sterne using the inducible plasmid pUTE657 to see if increasing overall FtsZ levels would change antibiotic resistance. Although we saw induction of *ftsZ* expression (Supplementary Figure 6A), there was no measurable effect on resistance to LL-37 (Supplementary Figure 6B) or penicillin (data not shown). We conclude there are changes in cell envelope morphology and/or composition including increased hydrophobicity and thinner cell walls in Δ*clpX*, and the extent of some of these differences, specifically cell wall thickness and cell division, is influenced by media composition.

## Discussion

We show that antibiotic resistance is dependent on the formation of the ClpXP protease and that both ClpP subunits, ClpP1 and ClpP2, contribute in a non-redundant manner as loss of either subunit by itself is enough to increase antibiotic sensitivity. However, although both ClpP subunits are necessary for resistance to the vast majority of cell-envelope targeting antimicrobials we tested, the phenotypes in the Δ*clpP1* or Δ*clpP2* mutants were never as strong as Δ*clpX* and, in the case of vancomycin, loss of neither *clpP1* nor *clpP2* had a significant effect on antibiotic susceptibility. In the *G. mellonella* infection model, loss of a single *clpP* subunit also had no effect on overall virulence. There are several conceivable explanations for this. While the *clpP* subunits cannot entirely compensate for another, it is possible they have some overlapping function, and this is sufficient to abrogate any virulence defect in a single mutant and lessen the severity of antibiotic resistance phenotype. It is also possible that even though ClpXP protease formation is essential for resistance to cell-wall targeting antibiotics, ClpX chaperone activity plays a role in virulence. Lastly, loss of either ClpP subunit could alter the stoichiometry of ClpXP formation, even if it does not entirely disrupt it, resulting in variable phenotypes depending on the nature of the stress.

Although it is relatively rare for bacteria to possess two *clpP* subunits, there are examples of *clpP1* and *clpP2* genes found in *Mycobacterium tuberculosis*, *Chlamydia trachomatis*, *Listeria monocytogenes, Pseudomonas aeruginosa*, and *Clostridioides difficile* (Akopian et al., 2012; Dahmen et al., 2015; Hall et al., 2017; Lavey et al., 2019; Pan et al., 2019). Although these organisms all possess two *clpP* genes, there are notable differences. For example, in *M. tuberculosis*, *clpP* genes are co-transcribed on the same operon (Personne et al., 2013), whereas in other species they are regulated independently. In *B. anthracis*, *clpP1* is regulated as a single gene whereas *clpP2* is found as part of a putative two-gene operon. The CtsR repressor is a common regulator of *clpP* expression in gram-positive bacteria (Frees et al., 2007) and the promoter region of the *clpP1* gene in both *B. thuringiensis* (Fedhila et al., 2002) and *B. anthracis* contains the same CtsR consensus sequence (5′-ggtcaataaaggtcaaa-3′). A gene encoding a CtsR homolog is also found in both species (Fedhila et al., 2002). No CtsR consensus region is found in the promoter region of *clpP2* and instead gene expression is closely tied to ClpX with *clpP2* levels increasing over 300-fold in the Δ*clpX* mutant. Levels of homology between the ClpP subunits also differs. In *B. anthracis*, the two ClpP subunits have high levels of similarity, as do ClpP1 and ClpP2 in *C. difficile* and *M. tuberculosis.* In contrast, ClpP1 and ClpP2 are highly dissimilar in *L. monocytogenes* and *C. trachomatis* suggesting a non-paralogous relationship (Pan et al., 2019). Lastly, interactions between ClpP subunits differ between species. *M. tuberculosis* and *C. trachomatis* require ClpP1 and ClpP2 to interact to form an active ClpP1P2 heterocomplex and in *L. monocytogenes* the ClpP1P2 heterocomplex enhances proteolytic activity (Akopian et al., 2012; Dahmen et al., 2015; Pan et al., 2019). Conversely, in *C. difficile* the ClpP1 and ClpP2 subunits function independently as does ClpP1 in *P. aeruginosa* (Hall et al., 2017; Lavey et al., 2019). Further experiments are needed to better understand relative contributions of each of the ClpP subunits in *B. anthracis* as well as whether the subunits interact with ClpX independently or whether a ClpP1P2 complex is required.

The importance of the ClpXP protease is well-documented in many bacterial species, but much remains to be discovered regarding the specific impacts on cellular physiology (Frees et al., 2007; Brötz-Oesterhelt and Sass, 2014). Due to their nature as global regulators, deleting *clpX, clpP1* and/or *clpP2* will have pleiotropic effects on the cell and it is unlikely that there is one single target that accounts for the phenotypes seen with cell-envelope targeting antibiotics, but rather multiple changes are occurring as part of the cell envelope stress response. Our previous study identified several sigma factors within the regulatory network of ClpX, including σ^*M*^, a known regulator of the cell envelope stress response system in *B. subtilis* that is induced by exposure to cell wall antibiotics such as vancomycin (Jordan et al., 2008; Claunch et al., 2018). We focused on changes to the cell envelope in large part because of the strong connection between loss of *clpX* and increased susceptibility to antibiotics that target it. We found no difference in cell charge, response to hypoosmotic stress, or autolytic activity despite trying autolysis assays under several different conditions in this and a previous study (Claunch et al., 2018). This is consistent with results in *S. aureus* where, despite the accumulation of autolytic enzymes in *clpX* and *clpP* mutants, no change in autolytic activity is observed in methicillin-susceptible *S. aureus* strains (Bæk et al., 2014). However, we did see a notable increase in Δ*clpX* hydrophobicity. Hydrophobicity is important for adhesion and biofilm formation, and it is linked to changes in cell wall composition particularly lipoteichoic acid (Courtney et al., 2009), but its role in antibiotic resistance is less clear. Increased resistance to cell-envelope targeting antimicrobials has been associated with increased and decreased hydrophobicity depending on the organism and antimicrobial being tested (Davies et al., 1996; Clarke et al., 2007; Lather et al., 2016). Therefore, while the increased hydrophobicity of Δ*clpX* indicates changes in cell wall composition, the extent to which this change is influencing antibiotic sensitivity is difficult to determine. We also see a significant decrease in overall cellular density of the *clpX* mutant. Unlike the phenotypes we observed with cell-envelope targeting antibiotics cellular density may not be solely dependent on ClpXP activity as there was still partial complementation with the *clpX*^*I*264*E*^ plasmid.

Although multiple factors could contribute to changes in cellular density, cell wall composition is certainly one of them (Makinoshima et al., 2002). Examination of the wild type and Δ*clpX* cell wall by TEM revealed several distinct differences, including thinner cell walls in Δ*clpX*. Media type significantly impacted the extent of these changes. When Δ*clpX* cells are grown in the mammalian cell culture media RPMI, they not only exhibit thinner cell walls than Δ*clpX* cells grown in BHI, but also significant cell division defects. It is unclear what difference in BHI and RPMI is triggering these division defects, although we speculate that RPMI may be a more inherently stressful environment due to lower nutrient availability or the inclusion of some other factor not found in BHI and that this additional stress could be overwhelming the bacterium’s ability to compensate for the lack of ClpX. The importance of environment on phenotypic effects seen with *clpX* loss is not entirely unprecedented given that defects in septum formation in *S. aureus clpX* mutants are seen at 30^*o*^C but not 37^*o*^C (Jensen et al., 2019a). Future studies are needed to better elucidate the nature of these defects including how media composition, growing conditions, and growth phase influence their prevalence. Despite the dramatic effects of RPMI on Δ*clpX* these alone cannot account for the increased susceptibility with cell-envelope targeting antibiotics because penicillin, vancomycin and nisin were all tested in BHI and growth of Δ*clpX* is still significantly impaired.

Our study corroborates others that connect loss of *clpX* to a disruption in cell division and/or cell wall metabolism (Frees et al., 2007, 2014). In *S. aureus*, ClpX localizes to the septum and loss of *clpX* is associated with dysregulation of cell wall proteins (Jensen et al., 2019b; Kirsch et al., 2021) and defects in septum formation that appear to be due to ClpX chaperone activity (Jensen et al., 2019a). ClpX regulates FtsZ in several organisms by preventing FtsZ polymerization (Weart et al., 2005; Dziedzic et al., 2010; Sugimoto et al., 2010). In *B. subtilis*, stabilization of polymerized FtsZ led to extra FtsZ rings forming at the cell poles (Levin et al., 2001) and it is possible that loss of *clpX* in *B. anthracis* is leading to increased stability and formation of FtsZ rings that are contributing to the extra division sites in our cells. Interestingly, in *S. aureus* FtsZ ring formation is not affected by loss of *clpX* suggesting that the cell division defects are due to something else (Jensen et al., 2019a). Additional experiments are needed to determine whether ClpX-chaperone or ClpXP protease activity contributes to cell wall and cell division defects in *B. anthracis* as well as understanding how interactions between ClpX and FtsZ influence cell division. Despite the parallels between loss of *clpX* and defects in cell envelope morphology, there are also notable differences between species. Unlike in *B. anthracis* Sterne, loss of *clpX* in *S. aureus* leads to thicker cell walls and increased resistance to beta-lactam antibiotics (Bæk et al., 2014). Therefore, while the disruption of cell wall metabolism is a consistent theme, the phenotypes associated with *clpX* deletion may vary across species. We also recognize that loss of *clpX* has repercussions beyond just cell wall morphology and these may also contribute to antibiotic sensitivity and overall virulence. Regardless, a wealth of studies demonstrates the importance of the Clp system, both in regulating cellular physiology as well as potentially serving as pharmacological targets in the development of new antibiotics (Brötz-Oesterhelt and Sass, 2014), but it is also clear that much is yet to be learned. Future studies will focus on the roles of the different players in the Clp system as well as downstream targets in order to gain a better understanding of how interfering with this system impacts the bacterial cell.

## Data Availability Statement

The raw data supporting the conclusions of this article will be made available by the authors, without undue reservation.

## Author Contributions

SM conceived of the study, designed the experiments, and wrote the manuscript. LZ, CE, VD, QL, and DN performed the experiments and contributed to data analysis, experimental design, and manuscript editing. All authors contributed to the article and approved the submitted version.

## Conflict of Interest

The authors declare that the research was conducted in the absence of any commercial or financial relationships that could be construed as a potential conflict of interest.

## Publisher’s Note

All claims expressed in this article are solely those of the authors and do not necessarily represent those of their affiliated organizations, or those of the publisher, the editors and the reviewers. Any product that may be evaluated in this article, or claim that may be made by its manufacturer, is not guaranteed or endorsed by the publisher.

## Footnotes

1
www.rainbowmealworms.net
