# Supplementary material for: Loss of the ClpXP Protease Leads to Decreased Resistance to Cell-Envelope Targeting Antimicrobials in Bacillus anthracis Sterne
Source: Front Microbiol. 2021 Aug 23;12:719548. doi: 10.3389/fmicb.2021.719548 (PMC8419472; doi:10.3389/fmicb.2021.719548)
Supplement: Supplementary file 1 [file Data_Sheet_1.PDF]

Supplementary Material

1 Supplementary Methods

Cell Charge (Cytochrome C)

Bacterial cultures were washed in 20 mM MOPS buffer (pH 7.0), then centrifuged and resuspended in 20 mM MOPS buffer to an optical density of 5.0 at 600 nm. They were then incubated with 1 mg/mL cytochrome c for 10 minutes at room temperature in a total volume of 0.5 mL. After incubation, the samples were centrifuged at 16000 rpm for 3 minutes and the optical density of the supernatant was measured at 530 nm to measure residual cytochrome c.

Cell Lysis

Bacterial cultures were grown to an optical density of 0.4 at 600 nm and washed twice in 10mM sodium phosphate buffer (pH 7.0) and then resuspended in pure water either for the duration of the assay (osmotic lysis assay) or resuspended in ice-cold pure water for 10 minutes before being resuspended in sodium phosphate buffer with 0.05% Triton X-100 (Sigma) (autolysis assay) and incubated at 30°. Bacterial lysis was measured by optical density at 600 nm every 15 minutes and then expressed relative to starting OD to calculate percent intact cells at each time point.

Construction of *ftsZ* complementation plasmid, expression analysis, and antimicrobial assays

The *ftsZ* gene was amplified using primers ftsZ-EV_fwd-SalI 5′-ATGCGTCGACACGTGTATTCCGTTATTTATGGGATTA-3′ and ftsZ-EV_rev-SphI 5′-TGCAGCATGCGCAAAGAGCGGGATAGATTGAA-3′, subcloned into pUTE657 using SalI and SphI restriction endonucleases, transformed into MC1061F electrocompetent *E. coli* (Lucigen, Middleton WI, USA), and passaged through GM2163 before being transformed into *B. anthracis* Sterne. Expression of the *ftsZ* gene was induced with 1 mM IPTG and RNA was extracted and QPCR performed as described in the main methods with the exception that expression was normalized to *gyrB*. The following primers were used for QPCR: ftsZ-qpcrFwd 5′-CGTGCAACACAAGCGGCATC-3′; ftsZ-qpcrRev 5′- CGCCAATGTTAGAGGCATTCC GTG -3′; gyrB-Fwd 5′- GGTGTTGGGGCATCTGTAGT-3′; gyrB-Rev 5′- GTATTCCGGTTGCGGATTTA-3′. Antimicrobial susceptibility assays were performed as described in the main methods except that 1 mM IPTG was included in the overnight culture, log phase culture and assay media.

2 Supplementary Figures

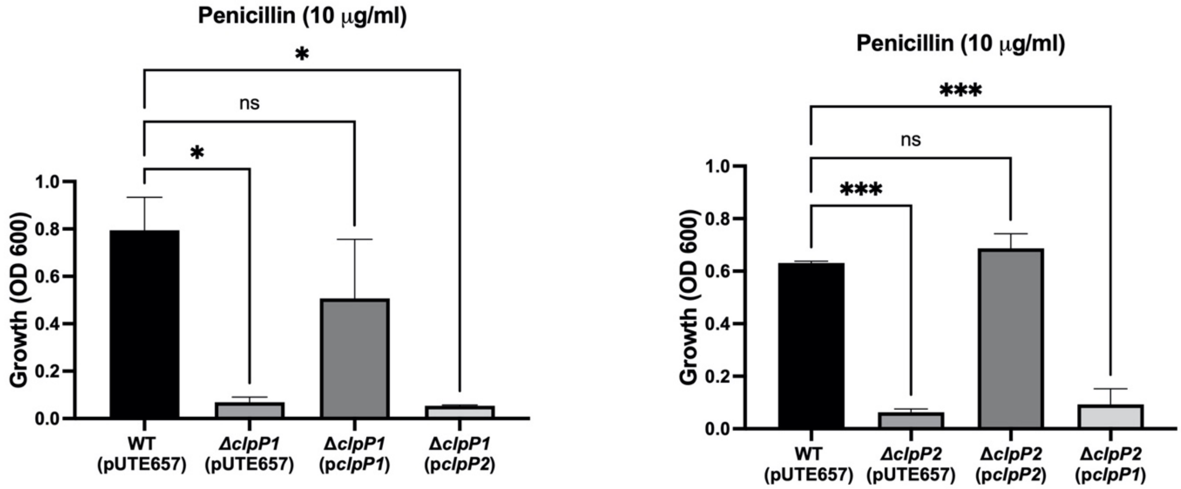

Figure S1. Swapping complementation plasmids is insufficient to restore resistance. Overnight growth in 10 µg/ml of penicillin of either wild-type *B. anthracis* Sterne (WT) containing the empty inducible plasmid (pUTE657) and A) Δ*clpP1* or B) Δ*clpP2* containing pUTE657 or the complementation plasmids p*clpP1* or p*clpP2* as indicated. *, p<0.05 and **, p < 0.001 compared to wild-type as determined by a one-way ANOVA followed by Dunnett's multiple comparison test. Data are presented as mean +/- SD of two independent experiments.

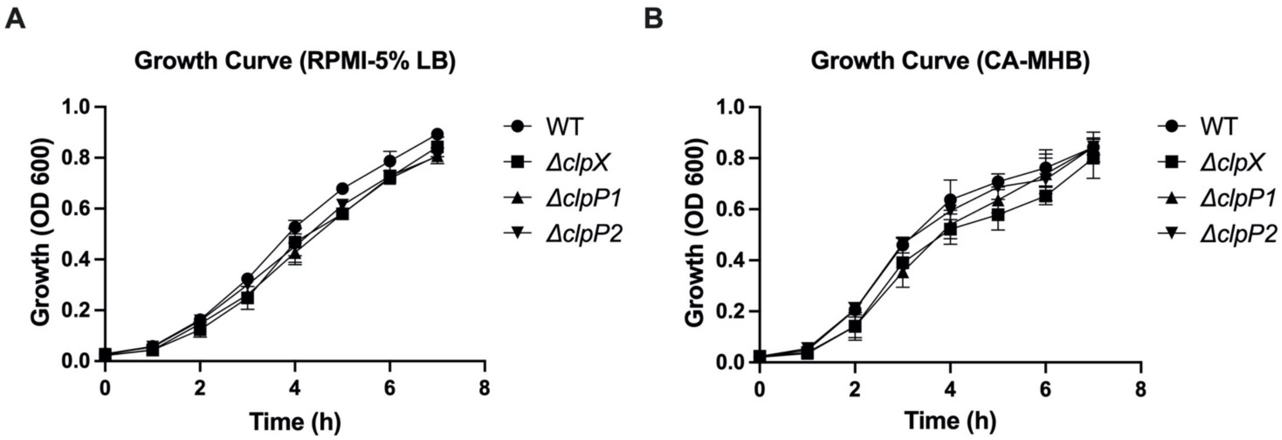

Figure S2. Growth of *B. anthracis* strains. Growth of wild-type *B. anthracis* Sterne (WT), Δ*clpX*, Δ*clpP1* or Δ*clpP2* in (A) RPMI-5% LB or (B) CA-MHB. Data represents median +/- SD of 2 independent experiments.

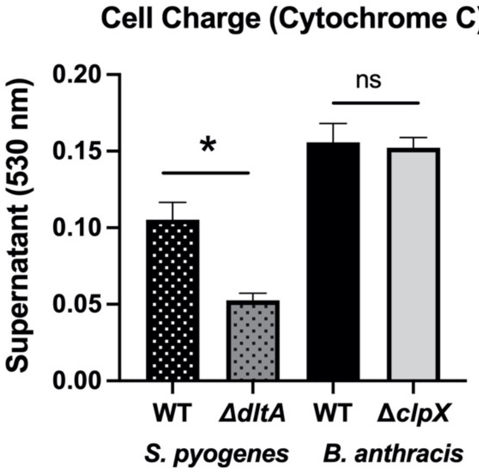

Figure S3. Cell charge using cytochrome C. Amount of cationic cytochrome C that sequesters in the supernatant after incubation with WT *S. pyogenes*, WT *B. anthracis* Sterne or their respective mutants Δ*dltA* and Δ*clpX*. *, $p < 0.05$ as determined by unpaired t-test between WT and mutant of each species. Data represents median +/- SD of 2 independent experiments.

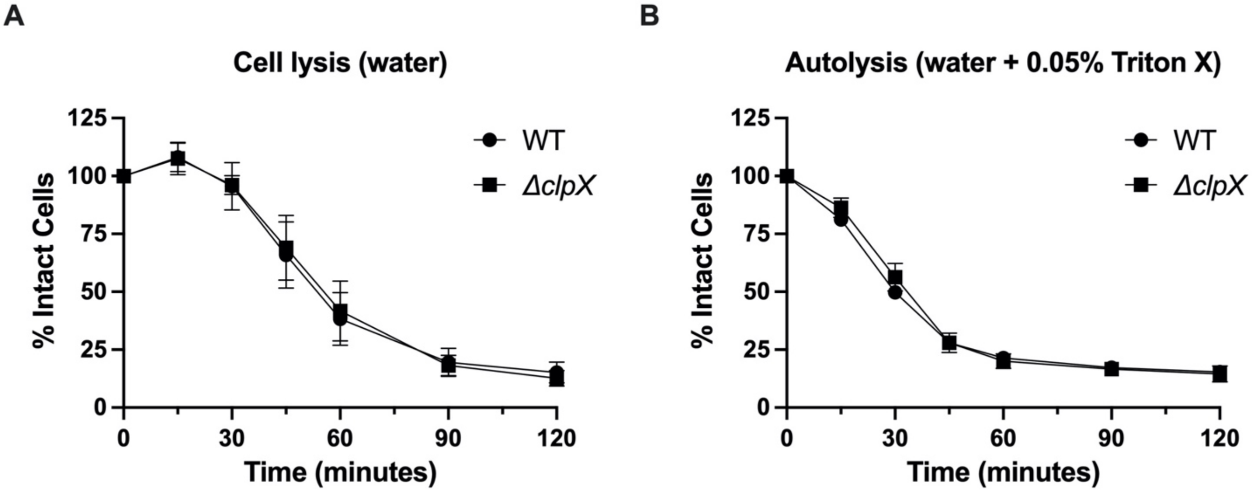

Figure S4. Cell lysis after osmotic shock. Percent intact cells as measured changes in optical density over time for (A) cells incubated in pure water and (B) cells incubated in cold water for 10 minutes followed by 0.05% triton-X. Data represents median +/- SD of 3 independent experiments.

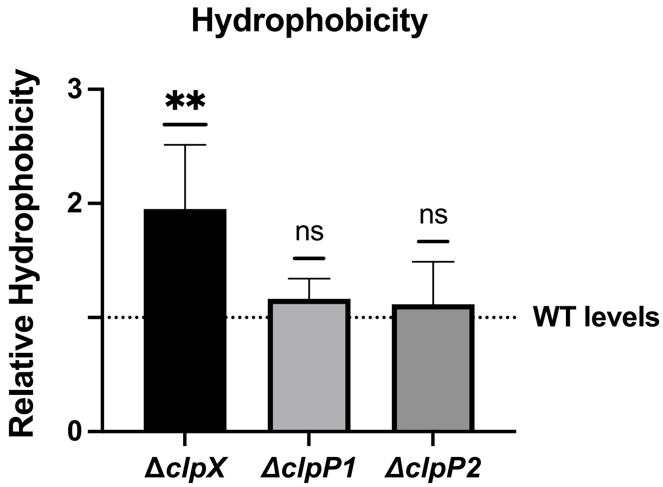

Figure S5. The *clpP* mutants do not exhibit significant differences in hydrophobicity. Amount of the Δ*clpX*, Δ*clpP1*, and Δ*clpP2* mutants that sequesters in n-hexadecane relative to the wildtype (WT) strain (dotted line). ** $p < 0.001$ as determined by a one-sample t-test. Data presented as mean +/- SD from 5 independent experiments.

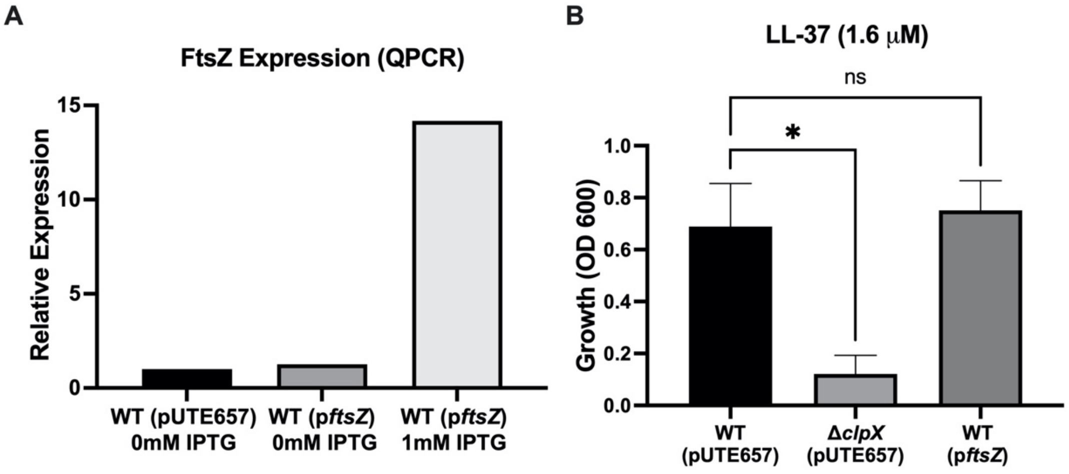

Figure S6. Overexpression of *fstZ* does not affect antibiotic resistance. (A) Expression levels of *ftsZ* in wild-type *B. anthracis* Sterne (WT) with empty pUTE657 plasmid and WT with the *ftsZ* expression plasmid (pFtsZ) with and without 1 mM IPTG. Data represents one independent sample. (B) Overnight growth of wild-type *B. anthracis* Sterne (WT) and Δ*clpX* mutant containing the empty plasmid (pUTE657) or the plasmid with the *ftsZ* gene *(*p*ftsZ)* after induction with 1 mM IPTG in the presence of 1.6 μM LL-37. Data represents median +/- SD of 2 independent experiments.
